# 2D-Perfusion Angiography Using Carbon Dioxide (CO2): A Feasible Tool to Monitor Immediate Treatment Response to Endovascular Therapy of Peripheral Arterial Disease?

**DOI:** 10.1007/s00270-020-02722-z

**Published:** 2020-12-16

**Authors:** Cornelia L. A. Dewald, Lena S. Becker, Sabine K. Maschke, Timo C. Meine, Bernhard C. Meyer, Frank K. Wacker, Jan B. Hinrichs

**Affiliations:** Institute for Diagnostic and Interventional Radiology, Hannover Medical School, Carl-Neuberg-Str. 1, 30625 Hannover, Germany

**Keywords:** 2D-perfusion angiography, Carbon dioxide, Negative contrast agent, Endovascular treatment, Stent, Peripheral arterial disease

## Abstract

**Purpose:**

Patients with peripheral arterial disease (PAD) or critical limb ischemia (CLI) require revascularization. Traditionally, endovascular therapy (EVT) is performed with iodinated contrast agent (ICM), which can provoke potential deterioration in renal function. CO2 is a safe negative contrast agent to guide vascular procedures, but interpretation of CO2 angiography is challenging. Changes in blood flow following iodine-aided EVT are assessable with 2D-perfusion angiography (2D-PA). The aim of this study was to evaluate 2D-PA as a tool to monitor blood flow changes during CO_2_-aided EVT.

**Material and Methods:**

2D-PA was performed before and after ten EVTs (nine stents; one endoprosthesis; 10/2012–02/2020) in nine patients (six men; 65 ± 10y) with Fontaine stage IIb (*n* = 8) and IV (*n* = 1). A reference ROI (ROI_INFLOW_) was placed in the artery before the targeted obstruction and a target ROI (ROI_OUTFLOW_) distally. Corresponding ROIs were used pre- and post-EVT. Time to peak (TTP), peak density (PD) and area under the curve (AUC) were computed. The reference/target ROI ratios (TTP_OUTFLOW_/TTP_INFLOW_; PD_OUTFLOW_/PD_INFLOW_; AUC_OUTFLOW_/AUC_INFLOW_) were calculated.

**Results:**

2D-PA was technically feasible in all cases. A significant increase of 82% in PD_OUTFLOW_/PD_INFLOW_ (0.44 ± 0.4 to 0.8 ± 0.63; *p* = 0.002) and of 132% in AUC_OUTFLOW_/AUC_INFLOW_ (0.34 ± 0.22 to 0.79 ± 0.59; *p* = 0.002) was seen. A trend for a decrease in TTP_OUTFLOW_/TTP_INFLOW_ was observed (− 24%; 5.57 ± 3.66 s–4.25 ± 1.64 s; *p* = 0.6).

**Conclusion:**

The presented 2D-PA technique facilitates the assessment of arterial flow in CO2-aided EVTs and has the potential to simplify the assessment of immediate treatment response.

## Introduction

Peripheral arterial disease (PAD) is a major public health problem with rising prevalence and considerable socioeconomic impact [1–3]. Current guidelines recommend an “endovascular first” strategy for stenosed or occluded vessels in patients with claudication and critical limb ischemia, which leads to an increase in endovascular therapy (EVT) [1, 2, 5, 6]. Coexisting renal, diabetic, vascular and other chronic comorbidities are common in patients with PAD, which can complicate disease management [2, 7, 8]. Carbon dioxide (CO2) has long been acknowledged as a safe intravascular, negative contrast agent to guide vascular procedures in patients with chronic kidney disease (CKD), hypersensitivities to iodinated contrast agent (ICM) or hyperthyroidism [1, 4, 9–11]. Nevertheless, CO2 is reported to be underutilized in the past [12]—mainly due to the discrepancy between CO2 and ICM regarding application and image analysis. The unsteady image quality in this infrequently used technique can limit the interpretation of contrast flow changes following EVT. This might negatively affect EVT with CO2, as the angiographic endpoint in EVT depends on the visual comparison of blood flow before and after revascularization.

2D-perfusion angiography (2D-PA) is a newly available technique for the assessment of blood flow and tissue perfusion. This technique relies on dedicated post-processing of standard digital subtraction angiography (DSA) images and computes time–density curves, which represent the flow of contrast agent within a region of interest (ROI) throughout the entire DSA run [2, 18–22]. Thus, it allows us to assess the perfusion distal to occluded vessels [2, 13–17] before, during and after endovascular revascularization. This might offer an opportunity to predict and evaluate clinical outcomes. 2D-PA used in CO2-guided interventions might increase the diagnostic confidence and thus support the visual interpretation. The aim of this study was to evaluate the feasibility of 2D-PA as a tool to monitor perfusion changes after CO2-aided EVT in patients with PAD.

## Material and Methods

### Patient Population

In total, 857 diagnostic and interventional DSA of the lower extremity, performed at our institution between 10/2014 and 02/2020, were screened for CO2-aided imaging. Out of these, we included all consecutive studies with paired pre- and post-interventional DSA series performed with CO2. In total, ten interventions in nine patients with PAD admitted for EVT of the pelvic/lower extremity arteries were assessed. Indications for CO2 as contrast agent were severe CKD with preserved urine excretion in all patients (*n* = 9/9; 100%) and additional allergy to ICM and hyperthyroidism in one patient each (*n* = 1/9; 11%).

### Angiography

The DSA images were acquired using a monoplane, ceiling-mounted angiographic system equipped with a 30 × 40 cm flat-panel detector (Artis Q^®^ or Artis pheno, Siemens Healthcare, Forchheim, Germany). Intra-arterial CO2 was administered using a dedicated angiography set (CO2-Angioset nach Schmitz-Rode/Alzen, Optimed, Ettlingen, Germany) via transfemoral approach.

For each DSA of the pelvic or the femoral arteries, 60–80 ml of pure CO2 was administered with a constant injection pressure of 1.3 bar [21]. Imaging parameters included a tube voltage of 60–80 kV and a tube current of 50–450 mA. Images were acquired at 7.5 frames/second with a range of 10–20 s, commencing at the same time as contrast injection. Interventions were performed with FOV adjusted to the investigated region using the 30 × 40 cm detector with collimation. Spatial resolution is 125 µm.

In patients with a known severe iodinated contrast agent allergy, CO2 was the only contrast agent used. In patients without absolute contraindications for ICM, hybrid angiographies were performed at the discretion of the interventionalist (*n* = 7). In hybrid angiography, ICM-based images were acquired in between a mainly CO2-aided intervention if image quality was insufficient and the interventionalist was not comfortable with sole CO2-based imaging.

Residual stenosis of < 30% of the vessel diameter after EVT by visual assessment of the performing interventionalist was set as the endpoint [2].

### Image Analysis

Post-processing of the original angiographic DICOM dataset was computed on a dedicated workstation using a commercially available software package (syngo X Workplace^®^VD10A, iFlow^®^, Siemens Healthcare). One reference region of interest (ROI) was placed in the artery proximal to the vascular lesion (to assess the arterial inflow; ROI_INFLOW_), and a second ROI (ROI_OUTFLOW_) was placed distal to the vascular lesion to assess arterial outflow. All ROIs were fitted to at least 2/3 of the vessel diameter. As shape and positioning of the ROIs might influence the results [14], we used circular ROIs on the pre-interventional DSA, which were semiautomatically copied to the corresponding DSA following revascularization in order to ensure that shape and position of the ROIs were comparable.

Numeric density values for the time to peak (TTP), peak density value (PD) and area under the time–density curve (AUC) were computed [2, 13–18]. The following ratios were calculated pre- and post-intervention: TTP_OUTFLOW_/TTP_INFLOW_, PD_OUTFLOW_/PD_INFLOW_ and AUC_OUTFLOW_/AUC_INFLOW_. Comparison of the pre- and post-intervention 2D-perfusion parameter ratios was performed. Patient motion during an angiographic run was analyzed, based on visual impressions.

### Statistical Analysis

Descriptive statistical analyses of the patient demographics and angiographic data were performed. Comparisons between pre- and post-intervention data were tested using pairwise Wilcoxon signed-rank test. A *p* value of  ≤ 0.05 was defined as statistically significant. Statistical analyses were computed using commercially available software (JMP 14, SAS Institute, JMP Office Germany, Böblingen, Germany).

## Results

In total, this study included ten interventions in nine patients (six men, three women; mean age 65 ± 10 years), who were admitted for CO2-aided EVT due to clinically relevant PAD (Fontaine stage IIB (9/10) and IV (1/10)) of the pelvis (6/10; 60%) or lower extremity (4/10; 40%). In 7/9 patients, leading indication for EVT was intermittent claudication. In total, 5/9 patients (55%) had a previous kidney transplant. Of those, 2/9 (22%) presented with acute renal deterioration due to PAD-associated perfusion changes in the transplanted kidney. All patients suffered from CKD stage 4 (*n* = 7/9; 78%) or stage 5 (*n* = 2/9; 22%). In addition to CKD, one patient (1/9; 11%) suffered from a severe allergy to ICM and another patient (1/9; 11%) presented with hyperthyroidism. For detailed patient characteristics, refer to Table 1.

**Table 1:** Patient demographics and clinical parameters

Parameters	Values
Number of patients, n	9
Male sex, n (%)	6/9 (67%)
Age, years*	65 ± 10
Body Mass index*	25.8 ± 4.6
Kidney transplantation, n (%)	5/9 (55%)
Arterial hypertension, n (%)	9/9 (100%)
Diabetes mellitus, n (%)	4/9 (44%)
Coronary artery disease, n (%)	6/9 (67%)
Anemia, n (%)	7/9 (78%)
Smoker, n (%)	2/9 (22%)
Hyperthyroidism, n (%)	1/9 (11%)
Contrast agent allergy, n (%)	1/9 (11%)
*Chronic kidney disease, n (%)*	9/9 (100%)
Disease stage 4	7/9 (78%)
Disease stage 5	2/9 (22%)
Dialysis (with residual renal excretion)	2/9 (22%)
*Peripheral arterial disease, n (%)*	9/9 (100%)
Fontaine stage IIb	8/9 (89%)
Fontaine stage IV	1/9 (11%)
Pre-interventional walking distance, m*	123 ± 108
Mediasclerosis, n (%)	2/9 (22%)

*Shown are mean values and standard deviations

Revascularization (nine stents, one endoprosthesis) was successfully achieved in all patients (common iliac artery (*n* = 6), superficial femoral artery (*n* = 4)). In 7/10 interventions, hybrid angiography was performed. In 3/10 interventions (performed in 2/9 patients), administration of additional ICM was not necessary. No patient had to be excluded due to motion artifacts. In the two patients admitted with acute transplant dysfunction, serum creatinine decreased and eGFR increased following revascularization (serum creatinine of patient 1: 406 μmol/l (eGFR 15 ml/min) before and 195 μmol/l (eGFR 33 ml/min) 1 week after EVT; serum creatinine of patient 2: 338 μmol/l (eGFR 15 ml/min) pre- and 303 μmol/l (eGFR 18 ml/min) 1 week post-intervention) (Table 2).

**Table 2:** Procedure characteristics

Parameters	Values
*Number of interventions*	10
Stent, n (%)	9/10 (90%)
Endoprosthesis, n (%)	1/10 (10%)
*Iliac artery, n (%)*	6/10 (60%)
Common iliac artery	4/10 (40%)
Common and external iliac artery	2/10 (20%)
*Femoral artery, n (%)*	4/10 (40%)
Primary technical success, n (%)	10/10 (100%)
Irradiation time, min*	8.46 ± 4.02
Area dose product (cGy x cm^2^)*	4274.47 ± 4368.5
Iodinated contrast medium, ml*	43.2 ± 59.73
DSA iliac artery	52.83 ± 76.68
DSA femoral artery	28.75 ± 20.97
*Creatinine (µmol/l)*	
Before revascularization*	234.6 ± 86.19
Following revascularization*	233.11 ± 74.51
*p *value	0.57**
*Estimated glomerular filtration rate (ml/min)*	
Before revascularization*	24.3 ± 7.78
48 h following revascularization*	24.78 ± 7.89
*p *value	0.63**
1 week following revascularization*	25 ± 7.29
*p *value	0.35**

*Shown are mean values and standard deviations

**Statistically not significant

**Fig. 1:**
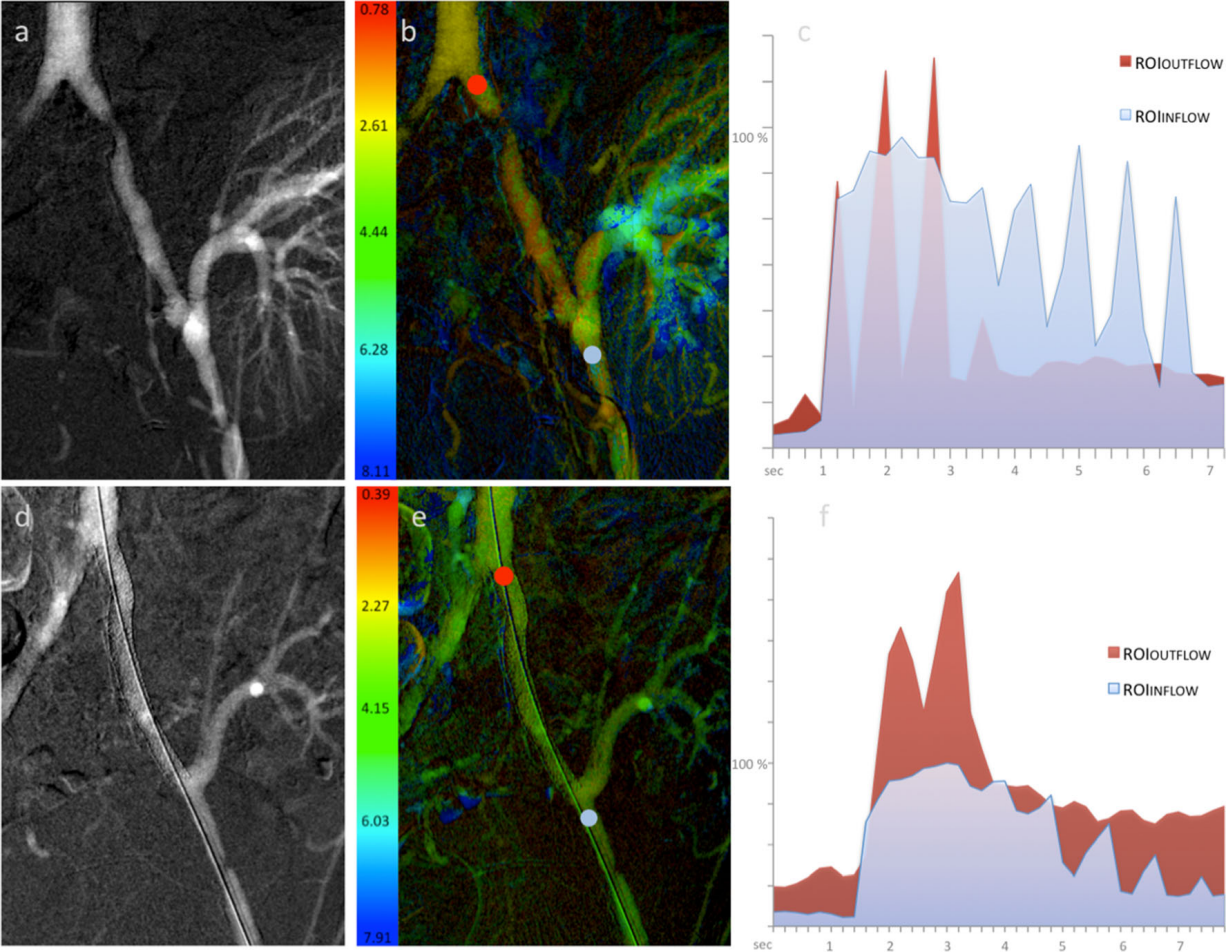
Example of ROI placement and 2D-PA parameters changes following iliac endovascular therapy. Example of CO_2_-aided DSA and color-coded 2D-perfusion angiography image before (**A**, **B**) and after (**D**, **E**) stenting of a stenosis in the left iliac artery in a patient with kidney transplant. The ROI_INFLOW_ (blue) is placed in the distal iliac artery close to the tip of the catheter/sheath and the ROI_OUTFLOW_ (red) is placed distant to the stenosis/stent in the proximal iliac artery. Note the increased time density values and higher area under the curve within the ROI_OUTFLOW_ following revascularization (**F**) compared with (**C**). Note that the fluctuating curves are synchronized to the arterial blood pressure

**Fig. 2:**
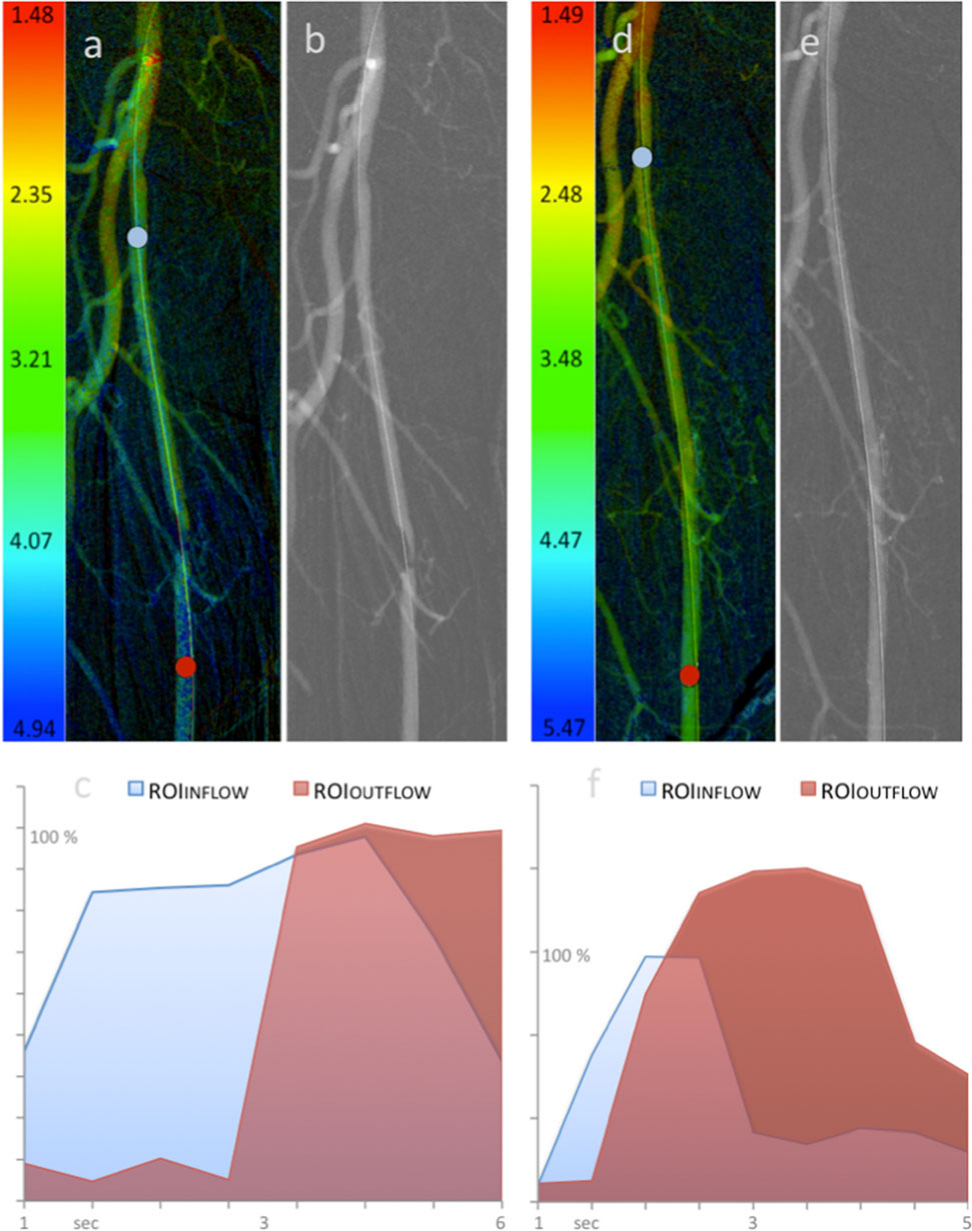
Example of ROI placement and 2D-PA parameters changes following femoral endovascular therapy. Example of CO_2_-aided DSA and color-coded 2D-perfusion angiography image before (**A**, **B**) and after (**D**, **E**) stenting of a stenosis in the right femoral artery. The ROI_INFLOW_ (blue) is placed in the proximal femoral artery to assess inflow and the ROI_OUTFLOW_ (red) is placed distant to the stenosis/stent. Note the increased time density values and higher area under the curve within the ROI_OUTFLOW_ following revascularization (**F**) compared with (**C**)

2D-PA facilitated detection of blood flow changes following recanalization in all procedures. After revascularization, PD_OUTFLOW_/PD_INFLOW_ significantly increased compared with the pre-interventional values (0.44 ± 0.4–0.8 ± 0.63; *p* = 0.002). Concordantly, AUC_OUTFLOW_/AUC_INFLOW_ presented significantly higher values following EVT (0.34 ± 0.22–0.79 ± 0.59; *p* = 0.002) indicating a higher amount of contrast passing through the treated lesion. A trend for a decrease in TTP_OUTFLOW_/TTP_INFLOW_ after the interventions was observed, but without reaching statistical significance (5.57 ± 3.66 s–4.25 ± 1.64 s; *p* = 0.6). Treatment results are summarized in Table 3; an example of 2D-PA is shown in Fig. 1 for iliac EVT and in Fig. 2 for femoral EVT.

**Table 3:** Changes in 2D-perfusion angiography parameters following revascularization

	Pre-intervention	Post-intervention	Difference (%)	*p* value
PD_OUTFLOW_/PD_INFLOW_	0.44 ± 0.4	0.8 ± 0.63	+ 0.36 (82%)	0.002
TTP_OUTFLOW_/TTP_INFLOW_	5.57 ± 3.66	4.25 ± 1.64	− 1.32 (24%)	0.6
AUC_OUTFLOW_/AUC_INFLOW_	0.34 ± 0.22	0.79 ± 0.59	+ 0.45 (132%)	0.002

Mean values with standard deviation and percentage difference between pre- and post-revascularization

*TTP* time to peak; *PD* peak density; *AUC* area under the curve

## Discussion

In this study, we evaluated the feasibility of 2D-PA as a tool to monitor perfusion changes following EVT in CO2-guided interventions. Angiographies during EVT in PAD have traditionally used ICM to accurately visualize the target arteries [12, 22]. However, ICM has significant associated risks, including hypersensitivity reactions, thyrotoxicosis and an increased danger of acute renal deterioration in patients with CKD. CO2 has been acknowledged as a low-risk contrast agent to guide vascular interventions in patients with CKD, hyperthyroidism or hypersensitivities to ICM [1, 4, 9–11]. Nonetheless, the image quality in CO2 angiographies is unsteady [1, 12, 23], and obtaining diagnostically valuable images in CO2 angiography can be more challenging than in conventional angiography [24], especially as CO2 angiographies are still less common compared with ICM. Generally, the assessment of blood flow and thus identification of occluded/stenotic arteries during EVT are highly subjective and operator-dependent [18]. The combination of the aforementioned difficulties in the evaluation of blood flow changes using CO2 might aggravate and lead to a significantly decreased diagnostic confidence.

2D-PA assigns density values to each area within an ROI and calculates mean values in each frame. These density values over time have been used to retrieve knowledge about flow rates, flow time and maximum values [2, 13–17]. In our study, we retrospectively assessed pre- and post-interventional blood flow through stenotic vessels using 2D-PA, evaluating it for possible assistance in CO2-based angiographies. As previously described, PD characterizes the maximum mean density within the ROI. The AUC is calculated from each density value at each time point using integration of successive values [2, 13–17]. The significant increases in PD_OUTFLOW_/PD_INFLOW_ and AUC_OUTFLOW_/AUC_INFLOW_ following EVT imply an intra-individually increased perfusion of the treated artery. TTP_OUTFLOW_/TTP_INFLOW_, equaling the time from the start of the DSA run until maximum density, decreased following EVT, as expected, indicating an accelerated speed of blood flow after removal of the intravascular obstruction. We attribute the lack of significance regarding TTP_OUTFLOW_/TTP_INFLOW_ to the small sample size. However, despite the preliminary trendsetting results, the definition of endpoints for a successful EVT based on 2D-PA requires further evaluation with larger study populations.

This study has several limitations. The major limitation is the small number of patients admitted for EVT that received CO2-aided DSA before and after revascularization. Therefore, larger studies are necessary to support our findings.

## Conclusion

Evaluation of blood flow changes following EVT in CO2-guided interventions using 2D-PA is feasible. This technique has the potential to objectify immediate treatment response and might result in the definition of objective, user-independent interventional endpoints.

## Abbreviations

2D-PA: 2D-perfusion angiography
AUC: Area under the time–density curve
CKD: Chronic kidney disease
CO2: Carbon dioxide
DSA: Digital subtraction angiography
eGFR: Estimated glomerular filtration rate
EVT: Endovascular therapy
ICM: Iodinated contrast medium
PAD: Peripheral arterial disease
PD: Peak density value
ROI: Region of interest
TTP: Time to peak
